# Structure of the IscB–ωRNA ribonucleoprotein complex, the likely ancestor of CRISPR-Cas9

**DOI:** 10.1038/s41467-022-34378-3

**Published:** 2022-11-07

**Authors:** Kazuki Kato, Sae Okazaki, Soumya Kannan, Han Altae-Tran, F. Esra Demircioglu, Yukari Isayama, Junichiro Ishikawa, Masahiro Fukuda, Rhiannon K. Macrae, Tomohiro Nishizawa, Kira S. Makarova, Eugene V. Koonin, Feng Zhang, Hiroshi Nishimasu

**Affiliations:** 1 Structural Biology Division, Research Center for Advanced Science and Technology, The University of Tokyo, Tokyo, Japan; 2 McGovern Institute for Brain Research at MIT, Massachusetts Institute of Technology, Cambridge, MA USA; 3 Howard Hughes Medical Institute, Cambridge, MA 02139 USA; 4 Broad Institute of MIT and Harvard, Cambridge, MA 02142 USA; 5 Department of Brain and Cognitive Science, Massachusetts Institute of Technology, Cambridge, MA 02139 USA; 6 Department of Biological Engineering, Massachusetts Institute of Technology, Cambridge, MA 02139 USA; 7 Komaba Institute for Science, The University of Tokyo, Meguro, Tokyo, Japan; 8 Graduate School of Medical Life Science, Yokohama City University, Yokohama, Japan; 9 National Center for Biotechnology Information, National Library of Medicine, National Institutes of Health, Bethesda, MD 20894 USA; 10 Department of Chemistry and Biotechnology, Graduate School of Engineering, The University of Tokyo, Tokyo, Japan; 11 Department of Biological Sciences, Graduate School of Science, The University of Tokyo, Tokyo, Japan; 12 Inamori Research Institute for Science, Kyoto, Japan

**Keywords:** Electron microscopy, RNA

## Abstract

Transposon-encoded IscB family proteins are RNA-guided nucleases in the OMEGA (obligate mobile element-guided activity) system, and likely ancestors of the RNA-guided nuclease Cas9 in the type II CRISPR-Cas adaptive immune system. IscB associates with its cognate ωRNA to form a ribonucleoprotein complex that cleaves double-stranded DNA targets complementary to an ωRNA guide segment. Although IscB shares the RuvC and HNH endonuclease domains with Cas9, it is much smaller than Cas9, mainly due to the lack of the α-helical nucleic-acid recognition lobe. Here, we report the cryo-electron microscopy structure of an IscB protein from the human gut metagenome (OgeuIscB) in complex with its cognate ωRNA and a target DNA, at 2.6-Å resolution. This high-resolution structure reveals the detailed architecture of the IscB–ωRNA ribonucleoprotein complex, and shows how the small IscB protein assembles with the ωRNA and mediates RNA-guided DNA cleavage. The large ωRNA scaffold structurally and functionally compensates for the recognition lobe of Cas9, and participates in the recognition of the guide RNA–target DNA heteroduplex. These findings provide insights into the mechanism of the programmable DNA cleavage by the IscB–ωRNA complex and the evolution of the type II CRISPR-Cas9 effector complexes.

## Abstract (web summary)

The cryo-EM structure of the IscB–ωRNA–target DNA complex provide insights into the ωRNA-guided DNA cleavage mechanism by IscB and the evolution of the type II CRISPR-Cas9 effector complexes

## Introduction

Cas9, a programmable RNA-guided DNA endonuclease, is the effector component of type II CRISPR-Cas adaptive immune systems. Cas9 associates with a CRISPR RNA (crRNA) and a *trans*-activating crRNA (tracrRNA) (or a synthetic single-guide RNA), and cleaves double-stranded DNA (dsDNA) targets complementary to the ~20-nucleotide (nt) crRNA guide segment derived from CRISPR spacers, using its RuvC and HNH nuclease domains^1,2^. In addition to the guide-target complementarity, Cas9 requires a specific nucleotide motif adjacent to target sequences, the protospacer adjacent motif (PAM), for DNA recognition. Some Cas9 proteins, such as *Streptococcus pyogenes* Cas9 (SpCas9), exhibit robust DNA cleavage activity in mammalian cells and have been harnessed for a variety of molecular technologies, including genome editing, base editing, and transcriptional regulation^3,4^.

IscB (insertion sequences Cas9-like OrfB) proteins are encoded in a distinct family of IS200/IS605 transposons and are likely ancestors of Cas9^5,6^. A recent study demonstrated that IscB is a programmable RNA-guided DNA endonuclease in the OMEGA (obligate mobile element-guided activity) systems^7^. While IscB and Cas9 share the RuvC-like nuclease domains containing three conserved catalytic motifs (RuvC-I–III), with an inserted Arg-rich segment known as the bridge helix (BH), and the HNH nuclease domain, IscB (~400 residues) is much smaller than Cas9 (~1000–1400 residues), mainly due to the lack of the α-helical recognition (REC) lobe (Supplementary Fig. 1a, b). Unlike Cas9, IscB contains an amino-terminal PLMP domain (named according to the corresponding distinct amino-acid motif). IscB associates with a ~200–400-nt non-coding RNA (referred to as ωRNA), which is substantially larger than the ~100-nt crRNA:tracrRNA guides of Cas9, to form a ribonucleoprotein complex that cleaves dsDNA targets complementary to a 5′ guide sequence in the ωRNA. IscB requires a target adjacent motif (TAM) for target DNA recognition, although its carboxy-terminal region lacks detectable sequence similarity with the equivalent PAM-interacting (PI) carboxy-terminal domain of Cas9. Among the diverse IscB orthologs, an IscB protein derived from the human gut metagenome (OgeuIscB) exhibits DNA cleavage activity in human cells, and potentially could be used as a new genome-editing tool^7^. Nevertheless, how the small IscB proteins assemble with their cognate ωRNAs to mediate RNA-guided DNA cleavage remains unknown.

## Results

### Overall structure of the IscB–ωRNA–target DNA complex

**Fig. 1:**
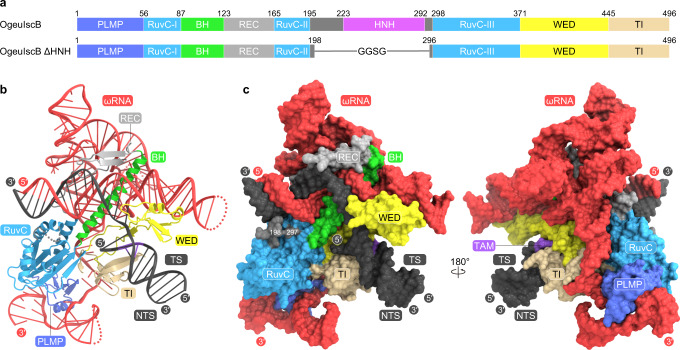
Overall structure of the IscB–ωRNA–target DNA complex. **a** Domain structures of the wild-type and ΔHNH mutant of OgeuIscB. BH, bridge helix. **b**, **c** Ribbon (**b**) and surface (**c**) representations of the IscB–ωRNA–target DNA complex. TS target DNA strand, NTS non-target DNA strand.

To prevent target DNA cleavage during our structural analysis, we co-expressed the OgeuIscB E193A/H247A mutant, in which the conserved catalytic residues of the two nuclease domains, E193 (RuvC) and H247 (HNH), are replaced with alanines, and its cognate ωRNA in *Escherichia coli* cells, and then purified the IscB–ωRNA complex. We reconstituted the IscB–ωRNA–target DNA ternary complex by mixing the purified IscB–ωRNA complex and the target DNA, and then attempted to determine the cryo-electron microscopy (cryo-EM) structure of the ternary complex. However, we failed to obtain a high-resolution density map, due to the orientation bias of the particles. Deletion of the HNH domain (residues 199–295), which is flexible and adopts multiple conformations in the Cas9 structure^8,9^, improved the quality of the cryo-EM images for the IscB–ωRNA–target DNA complex for an unknown reason. We determined the 2.6-Å resolution cryo-EM structure of the OgeuIscB mutant lacking the HNH domain (referred to as IscB for simplicity) in complex with a 233-nt ωRNA containing a 27-nt guide sequence and a partially double-stranded target DNA, consisting of a 49-nt target DNA strand and a 14-nt non-target DNA strand containing the GAAG TAM sequence (Fig. 1a, Supplementary Fig. 2a–i, Supplementary Table 1, Supplementary Movie 1).

The cryo-EM structure revealed that IscB comprises four globular domains, including the PLMP and RuvC domains, with the RuvC-I and RuvC-II motifs connected via the BH and a β-hairpin-containing linker (referred to as the REC linker), as well as the Wedge (WED) and TAM-interacting (TI) domains located similarly to the corresponding domains in Cas9 (Fig. 1b, c, Supplementary Movie 2). Nucleotides G( − 17)–C( − 1) in the 27-nt ωRNA guide segment were resolved in the density map, and base pair with nucleotides dG1–dC17 in the target DNA to form a 17-bp guide-target heteroduplex (Fig. 1b, c, Supplementary Fig. 3a). These structural observations indicate that the ~17-nt 5′ segment in the ωRNA functions as a guide sequence, consistent with a previous study showing that a 16-nt guide sequence is sufficient for the IscB-mediated DNA cleavage^7^. Nucleotides dA(−13)–dC(−1) and dG1*–dT13* in the target DNA form a 13-bp TAM-containing duplex (Fig. 1b, c, Supplementary Fig. 3b). Nucleotides G1–G206 in the ωRNA scaffold, except for the peripheral regions (U15–U29, U130–A141, and U173–A179), are also resolved in the density map (Supplementary Fig. 3c–e), providing high-resolution insights into the ωRNA architecture and the IscB–ωRNA interactions.

### IscB structure

**Fig. 2:**
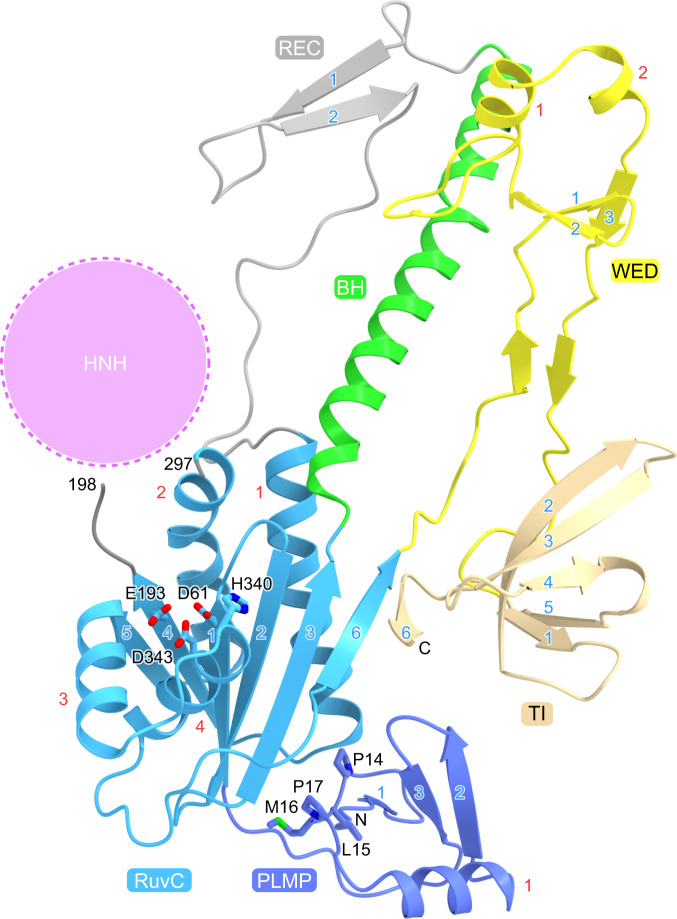
IscB structure. The conserved catalytic residues in the RuvC domain and the PLMP motif in the PLMP domain are shown as stick models. The possible location of the HNH domain is indicated by a dashed circle. The core α-helices and β-strands in each domain are numbered in red and blue, respectively.

The PLMP domain comprises a three-stranded mixed β-sheet and an α-helix (Fig. 2). The PLMP motif (residues 14–17) adopts a β-strand-like conformation and interacts with the RuvC domain and the ωRNA, stabilizing the IscB–ωRNA complex (Supplementary Fig. 4a, b). This observation can explain why mutations in the PLMP motif reduced the IscB-mediated DNA cleavage^7,10^. The RuvC domain adopts an RNase H fold and the configuration of its catalytic residues (D61, E193, H340, and D343) is similar to that in Cas9^11,12^ (Fig. 2, Supplementary Fig. 5). A density corresponding to a Mg^2+^ ion is present in the vicinity of D61 and E193 in the RuvC domain (Supplementary Fig. 4c), as observed previously in the Cas9 structure in the absence of the non-target DNA strand^13^. In contrast, two Mg^2+^ ions are bound to the RuvC domain of Cas9 in the presence of the non-target DNA strand, with its backbone phosphate group participating in the binding of the second Mg^2+^ ion^14^. Thus, the RuvC domain of IscB probably uses a catalytic mechanism similar to that of Cas9, in which the second Mg^2+^ ion binds to the RuvC domain upon the interaction with the non-target DNA strand. The WED domain contains a three-stranded antiparallel β-sheet and two α-helices (Fig. 2). The TI domain forms a five-stranded antiparallel β-barrel, with its carboxy-terminal β6 strand interacting with the β6 strand of the RuvC domain to form a seven-stranded β-sheet (Fig. 2). The Cas9 PI domain contains a core β-barrel structure similar to that of the IscB TI domain^11–13^, although the two domains lack detectable sequence similarity (Supplementary Fig. 5), suggesting that the Cas9 PI domain evolved from the IscB TI domain. A Dali search^15^ did not detect significant structural similarity between the PLMP/WED domains and any protein domains with known structures.

Although the present IscB structure lacks the HNH domain, structural prediction using AlphaFold2^16^ suggested that the HNH domain of IscB adopts a ββα-metal fold comprising a two-stranded antiparallel β-sheet and three α-helices, and is connected to the RuvC-II and RuvC-III motifs via two linkers (Supplementary Fig. 5), as in Cas9^11,12^. H247 of OgeuIscB is highly conserved among the IscB proteins (Supplementary Fig. 1b), and located in a similar position to that of the catalytic residue H840 in the Cas9 HNH active site (Supplementary Fig. 5), suggesting that the IscB HNH domain cleaves the target DNA strand via a Mg^2+^-dependent mechanism, as observed in Cas9^14^.

### ωRNA architecture

**Fig. 3:**
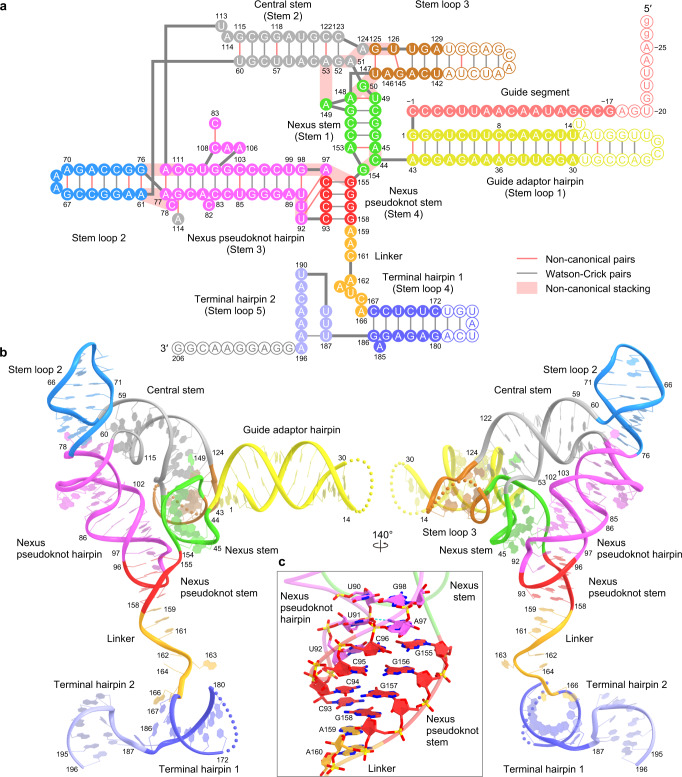
ωRNA architecture. **a**, **b** Schematic (**a**) and structure (**b**) of the ωRNA. Disordered nucleotides are indicated by circles with white backgrounds in **a** and dotted lines in **b**. **c** Close-up view of the nexus pseudoknot stem.

The ωRNA consists of a 27-nt guide segment (G( − 27)–C( − 1)) and a 206-nt ωRNA scaffold (G1–G206) (Fig. 3a, b, Supplementary Movie 3). The ωRNA scaffold comprises five stem loops (stem loops 1–5), four stems (stems 1–4), and a single-stranded linker (Fig. 3a, b). Stem loop 1 (guide adaptor hairpin) contains a 14-bp duplex (G1:A43–U14:A30) (Fig. 3a, b). Stem 1 (nexus stem) comprises a 5-bp duplex (A45:A153–U49:A148) with two non-canonical base pairs (A45:A153 and U49:A148) (Fig. 3a, b, Supplementary Fig. 6a). The guide adaptor hairpin connects the guide segment and the nexus stem. Stem 2 (central stem) contains a 9-bp duplex (G52:C123–U60:G115) with three non-canonical base pairs (A53:C122, U57:G118, and U60:G115), and stem loop 3 contains five base pairs (G125:U147–A129:U142) with two non-canonical base pairs (G125:U147 and U126:G145) (Fig. 3a, b). The nexus stem, central stem, and stem loop 3 form a three-way junction. A149 is flipped out from the nexus stem and interacts with A53 in the central stem, while A148-G50-U147 and G52-A51-A124-G125-U126 form a continuous base stack, thereby stabilizing the three-way junction (Supplementary Fig. 6a).

Stem loop 2 contains a 7-bp duplex (A61:G76–G67:A70) with two successive sheared A:G pairs (A61:G76 and A62:G75), and stem 3 (nexus pseudoknot hairpin) comprises a 12-bp distorted duplex (A77:A112–U90:G98) with a non-canonical A:A pair (A77:A112) and an internal loop (A106–A108) (Fig. 3a, b). Stem loop 2 coaxially stacks with the nexus pseudoknot hairpin through base stacking between the A61:G76 and A77:A112 pairs to form a contiguous helix, in which A112 adopts the *syn* conformation (Supplementary Fig. 6b). A114 in the central stem base pairs with G79 in the nexus pseudoknot hairpin to form an A114-G79:C111 base triple. In addition, C78 is flipped out from the nexus pseudoknot hairpin and interacts with A61 in stem loop 2. Notably, as predicted^7^, nucleotides C93–C96 in the nexus pseudoknot loop base pair with nucleotides G155–G158 downstream of the nexus stem to form stem 4 (nexus pseudoknot stem) (Fig. 3a, b). The C93:G158 and C96:G155 pairs stack with A159 and A97, respectively, and U92 base pairs with C96 to form a U92-C96:G155 base triple (Fig. 3c). The nexus pseudoknot hairpin extensively interacts with the nexus stem through their sugar-phosphate backbones (Supplementary Fig. 6c), thereby stabilizing the core of the ωRNA. The substitution of nucleotides C93–C96 with GGGG abolished the IscB-mediated genome editing in human cells (Supplementary Fig. 6d), confirming the functional importance of the pseudoknot structure in the ωRNA. The linker region (A159–A166) adopts a single-stranded conformation and connects the nexus pseudoknot stem, whereas the terminal hairpin regions consist of terminal hairpins 1 (C167–G186) and 2 (U187–A196) (Fig. 3a, b).

### IscB–ωRNA interactions

**Fig. 4:**
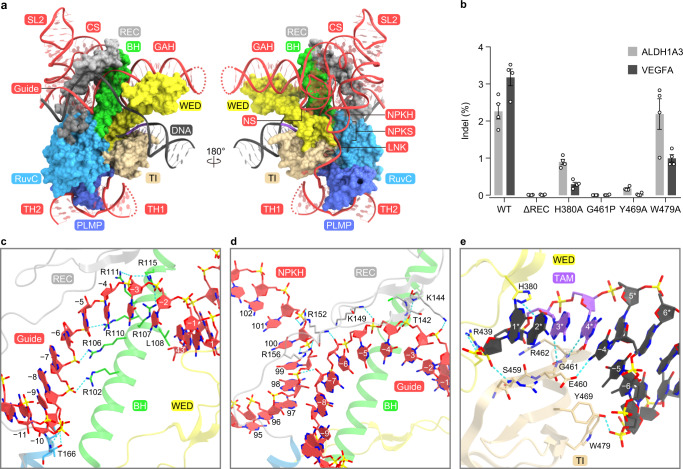
IscB–ωRNA–target DNA interactions. **a** Binding of the ωRNA and target DNA to the IscB protein. IscB is shown as a surface model. GAH guide adaptor hairpin, NS nexus stem, CS central stem, SL2 stem loop 2, NPKH nexus pseudoknot hairpin, NPKS nexus pseudoknot stem, LNK linker, TH1 terminal hairpin 1, TH2 terminal hairpin 2. **b** Indel activities of the wild-type (WT) and mutant IscB proteins. ΔREC is the REC mutant in which residues 129–144 were replaced with a GGGS linker. Data are mean ± s.e.m. (*n* = 4, biologically independent samples). The experiments were repeated four times with similar results. Source data are provided as a Source Data file. **c**–**e** Recognition of the seed region (**c**, **d**) and the TAM (**e**). Hydrogen bonds are shown as dashed lines.

IscB assembles with the ωRNA through extensive base-specific and nonspecific contacts (Fig. 4a, Supplementary Fig. 7). The guide adaptor hairpin and nexus stem are recognized by the BH and the WED domain (Fig. 4a, Supplementary Fig. 7). In particular, A43, the last nucleotide of the guide adaptor hairpin, stacks with L108, whereas the backbone phosphate between A43 and C44 interacts with H105, inducing a kink between A43 (guide adaptor hairpin) and C44 (nexus stem) (Supplementary Fig. 8a). The nucleobase of C44 is sandwiched between those of A45 and G154, and is recognized by R376, A378, and C379 through multiple hydrogen-bonding interactions. In turn, G154 stacks with Y101 and hydrogen bonds with H105 and Q377 (Supplementary Fig. 8a). The central stem and nexus pseudoknot hairpin extensively interact with the REC linker (Fig. 4a, Supplementary Fig. 7). C82 is flipped out from the nexus pseudoknot hairpin, and forms stacking and hydrogen-bonding interactions with R152 and N154/N155, respectively (Supplementary Fig. 8b). The nexus pseudoknot stem is recognized by the BH and the RuvC/WED domains, through the backbone interactions with nucleotides G155–G158 (Fig. 4a, Supplementary Fig. 7). G156 and G157 also form base-specific hydrogen bonds with R376 and H374, respectively (Supplementary Fig. 8c). The linker region is bound to a surface groove between the WED/TI and PLMP/RuvC domains (Fig. 4a, Supplementary Fig. 7). The nucleobases of A159 and A160/C161 hydrogen bond with R373 and N488/N489, respectively, while R487 intercalates between C161 and A162 (Supplementary Fig. 8c). The terminal hairpins are recognized by the PLMP domain, mainly through sequence-independent interactions (Fig. 4a, Supplementary Fig. 7). Stem loops 2 and 3 do not directly contact the IscB protein. Deletion of the REC linker reduced the structural integrity and the DNA cleavage activity of the IscB–ωRNA complex in vitro (Supplementary Fig. 9a–c), and abolished the IscB-mediated genome editing in human cells (Fig. 4b). Together, these structural findings revealed how OgeuIscB assembles with its cognate ωRNA to form a ribonucleoprotein complex.

### Target DNA recognition

The 17-bp guide RNA-target DNA heteroduplex is accommodated within a central channel formed by the BH, the REC linker, and the RuvC/WED domains, and is recognized by the protein primarily through sugar-phosphate backbone interactions (Fig. 4a, Supplementary Fig. 7). Nucleotides A( − 8)–C( − 1) in the ωRNA guide segment are anchored through interactions between their backbone phosphate groups and a cluster of Arg residues in the bridge helix (Fig. 4c). Thus, similarly to the ~10-nt seed region in the Cas9 guide RNA^17^, nucleotides (−8)–(−1) in the ωRNA guide segment could serve as a seed for target DNA recognition. The 2′-OH of A( − 7) in the guide segment interacts with the phosphate group between G98 and U99 in the ωRNA nexus pseudoknot hairpin (Fig. 4d), indicating that the ωRNA scaffold interacts with the ωRNA guide segment and contributes to the heteroduplex recognition. The phosphate group between dC(−1) and dG1 in the target DNA interacts with the main-chain amide group of A382 in the WED domain (Supplementary Fig. 8d), facilitating the guide-target hybridization, as observed in Cas9^13^. To examine the effect of mismatches between the ωRNA guide and the target DNA on IscB-mediated DNA cleavage, we performed in vitro DNA cleavage experiments, using the IscB–ωRNA complex and mismatch-containing DNA targets. TAM-proximal mismatches (positions 1–14), but not TAM-distal ones (positions 15–16), abolished the target DNA cleavage by IscB (Supplementary Fig. 9d), indicating the importance of the TAM-proximal ~14 base pairs in the formation of a guide-target heteroduplex susceptible to IscB-mediated DNA cleavage.

OgeuIscB recognizes the NNRR (N is A, T, G, or C, and R is A or G) sequence in the non-target DNA strand as the TAM^7^. In the present structure, the DNA duplex with the GAAG TAM is bound to a surface groove between the WED and TI domains (Fig. 4a). The β2–β3 hairpin in the TI domain is inserted into the major groove of the TAM duplex (Fig. 4e). The dG1* nucleobase does not contact the protein, consistent with the lack of preference for the first TAM nucleotide. The N3 of dA2* and the O2 of dT(−2) hydrogen bond with H380 and K381, respectively (Fig. 4e), which explains the slight preference of OgeuIscB for the second A in the TAM^7^. Indeed, the H380A mutation reduced the IscB-mediated DNA cleavage (Fig. 4b, Supplementary Fig. 9c). Notably, the N7 atoms of dA3* and dG4* form hydrogen bonds with the main-chain amide groups of G461 (3.0 Å) and R462 (3.2 Å), respectively (Fig. 4e, Supplementary Fig. 8e). Manual modeling suggested that pyrimidine bases at positions 3 and 4 in the NNRR TAM would sterically clash with G461 and R462 (Supplementary Fig. 8f, g). The G461P mutation, which would disrupt a hydrogen bond with the third R nucleotide in the NNRR TAM (because a proline residue lacks the main-chain amide group), abolished the IscB-mediated DNA cleavage (Fig. 4b, Supplementary Fig. 9c). It is also possible that the G461P mutation eliminated the DNA cleavage activity due to the reduced local structural order, rather than the disruption of a hydrogen-bonding interaction. These observations can account for the preference of OgeuIscB for the third and fourth R nucleotides in the TAM. The N4 atom of dT(−4) hydrogen bonds with E460 (Fig. 4e), contributing to the fourth R recognition. In addition, the TAM duplex is recognized by the WED and TI domains through backbone interactions. Y469 and W479 do not recognize the TAM nucleobase, but interact with the sugar-phosphate backbone of dT(−6) and dT(−7) (Fig. 4e). The Y469A and W479A mutations abolished and attenuated the IscB-mediated DNA cleavage, respectively (Fig. 4b, Supplementary Fig. 9c). Together, these results revealed the mechanism of TAM recognition by OgeuIscB.

## Discussion

**Fig. 5:**
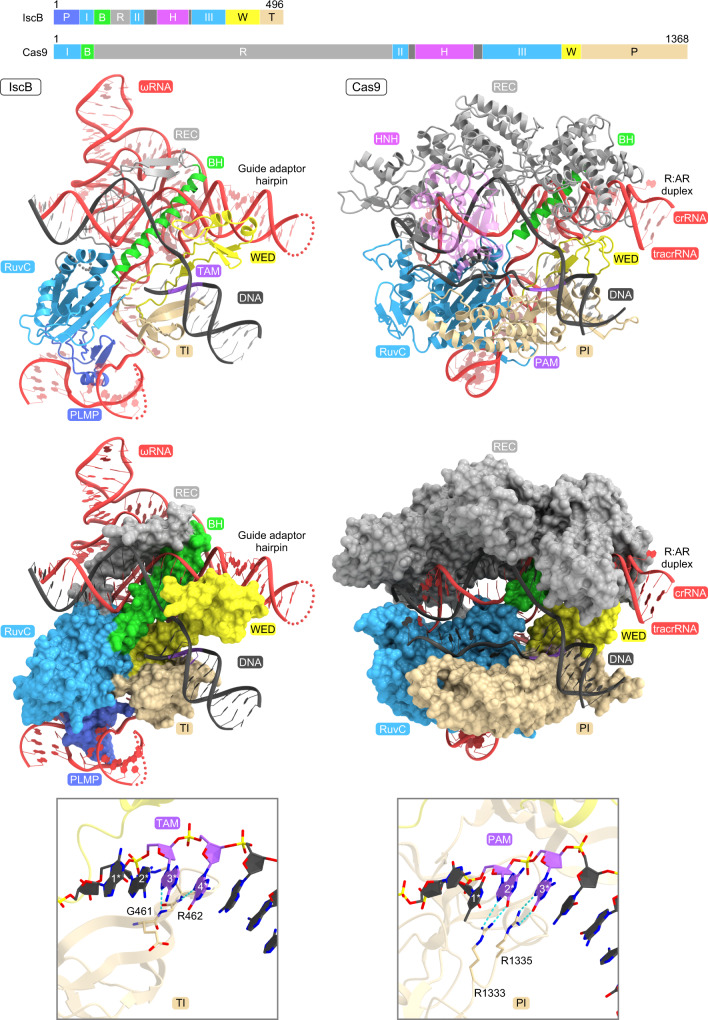
Structural comparison of IscB and Cas9. The structures of IscB and SpCas9 (PDB: 5F9R) were aligned, based on their RuvC domains. The HNH domain of Cas9 is colored semi-transparent magenta for clarity. P PLMP, I–III RuvC-I–III, B bridge helix, R REC, H HNH, W WED, T TI, P PI.

A structural comparison between IscB and Cas9 highlighted both the conservation and the differences in their RNA-guided DNA recognition mechanisms, thereby providing insight into the evolution of the type II CRISPR-Cas9 effector complex (Fig. 5). The guide segments of the IscB ωRNA and the Cas9 crRNA:tracrRNA similarly hybridize with the target DNA to form the heteroduplexes, which are recognized by their respective proteins in a sequence-independent manner. Furthermore, the seed region in the IscB and Cas9 guide segments is similarly anchored by the Arg-rich BH motifs. However, there are substantial differences between IscB and Cas9 in their heteroduplex recognition mechanisms. IscB uses the short REC linker (~40 residues) and the RuvC domain, together with the ωRNA scaffold, to recognize the ~14-bp heteroduplex. In contrast, Cas9 recognizes the ~20-bp heteroduplex through the large α-helical REC lobe (~600 residues) and the RuvC domain. Consistent with these structural differences, IscB and Cas9 require ~16- and ~20-nt guide sequences, respectively, with TAM/PAM-distal mismatches tolerated in both IscB (positions 15–16) and Cas9 (positions 18–20)^14^. Notably, the REC lobe of Cas9 senses mismatches within the heteroduplex and regulates the nuclease activities of the HNH and RuvC domains, thereby ensuring the fidelity of the target DNA cleavage^9,14^. These observations suggest that Cas9 proteins acquired REC lobes during their evolution from IscB, replacing the ancestral ωRNA scaffold, under selection pressure for fine-tuning the RNA-guided DNA cleavage mechanisms.

IscB and Cas9 recognize their cognate TAM and PAM, using the TI and PI domains with similar core β-barrel folds, respectively (Fig. 5). However, there are also intriguing differences in their TAM/PAM recognition mechanisms. OgeuIscB recognizes the NNRR TAM through the main-chain amide groups of G461 and R462 in the TI domain (Fig. 5). In contrast, Cas9 enzymes usually recognize their cognate PAMs through a variety of side-chain interactions in the PI domains. For example, SpCas9 recognizes the GG nucleotides in the NGG PAM, using the side chains of R1333 and R1335^13^ (Fig. 5). In addition, Cas9 enzymes interact with their cognate crRNA repeat:tracrRNA anti-repeat duplex (corresponding to the guide adaptor hairpin for IscB) through the REC and WED domains, which are structurally divergent among Cas9s^18^. These observations indicate that additional protein structural elements contribute to diversify the Cas9-mediated recognition of the guide RNAs and target DNAs, thereby enabling effective defenses against a wide variety of invading nucleic acids.

The present structure of the IscB–ωRNA–target DNA complex provides insights into the ancient programmable DNA cleavage mechanism catalyzed by the IscB–ωRNA ribonucleoprotein complex. Given the small size of IscB, the high-resolution structure reported here provides a framework for the future development of compact genome-engineering tools.

## Methods

### Preparation of the IscB–ωRNA–DNA complex

The gene encoding ωRNA (206-nt) with a 5′-guide sequence (27-nt) and OgeuIscB (residues 1–496) with an N-terminal His_14_-SUMO tag were cloned into the pETDuet-1 vector (Novagen) (Supplementary Table 2). The IscB mutants were prepared by a PCR-based method, and their sequences were confirmed by DNA sequencing. The IscB protein and the ωRNA were co-expressed in *Escherichia coli* Rosetta2 (DE3) (Novagen) by induction with 0.25 mM isopropyl β-D-thiogalactopyranoside (Nacalai Tesque) at 18 °C overnight. The *E. coli* cells were lysed by sonication in buffer A (20 mM Tris-HCl, pH 8.0, 20 mM imidazole, 300 mM NaCl, 3 mM 2-mercaptoethanol, 10% glycerol, 5 mM MgCl_2_, and 1 mM phenylmethylsulfonyl fluoride), and the lysate was clarified by centrifugation at 40,000 g. The supernatant was applied to Ni-NTA Superflow resin (QIAGEN) and the IscB–ωRNA complex was eluted with buffer B (20 mM Tris-HCl, pH 8.0, 300 mM imidazole, 300 mM NaCl, 3 mM 2-mercaptoethanol, 10% glycerol, and 5 mM MgCl_2_). The eluate was applied to a HiTrap Heparin column (GE Healthcare), equilibrated with buffer C (20 mM Tris-HCl, pH 8.0, 75 mM NaCl, 10% glycerol, 5 mM MgCl_2_, and 2 mM DTT). The bound protein was eluted with a linear gradient of 0.075–2 M NaCl. The peak fractions were collected and stored at −80 °C until use.

The IscB–ωRNA–target DNA complex was prepared for cryo-EM analysis according to the following procedure. A partially double-stranded DNA target was prepared by annealing a 49-nt target DNA strand (TS) (G1*–A49*) and a 14-nt non-target DNA strand (NTS) (G1*–C14*) containing a GAAG TAM at 95 °C for 1 min (Supplementary Table 2). The purified IscB–ωRNA complex (A_260_ of 3) was incubated with the target DNA (5 µM) at 37 °C for 40 min. The IscB–ωRNA–target DNA complex was purified by chromatography on a Superdex 200 Increase 10/300 column (GE Healthcare), equilibrated with buffer E (20 mM HEPES-NaOH, pH 7.0, 50 mM NaCl, 5 mM MgCl_2_, and 2 mM DTT). The peak fraction containing the IscB–ωRNA–target DNA complex was concentrated to an A_260_ of 12, using an Amicon Ultra-4 Centrifugal Filter Unit (MWCO 50 kDa) (Millipore).

### Cryo-EM grid preparation and data collection

Holey carbon grids (Au 300 mesh R0.6/1 grids, Quantifoil) were glow-discharged for 2 min. The IscB–ωRNA–target DNA complex was mixed with 0.1% LDAO (Anatrace), and the mixture (3 μL) was immediately applied to the freshly glow-discharged grids in a Vitrobot Mark IV (Thermo Fisher Scientific) at 4 °C, with a waiting time of 10 s and a blotting time of 4 s under 100% humidity conditions. The grids were plunge-frozen into liquid ethane cooled at liquid nitrogen temperature. The cryo-EM data were collected using a Titan Krios G3i microscope (Thermo Fisher Scientific), running at 300 kV and equipped with a Gatan Quantum-LS Energy Filter (GIF) and a Gatan K3 Summit direct electron detector. Micrographs were recorded at a nominal magnification of ×105,000, with a pixel size of 0.83 Å in a total exposure of 47.9 e^−^/Å^2^ per 48 frames, by the correlated double sampling mode. The data were automatically acquired by the image shift method using the EPU software (Thermo Fisher Scientific), with a defocus range of −0.8 to −2.0 μm, and 3,278 movies were acquired.

### Image processing

The data processing was performed with the cryoSPARC v3.3.1 software platform^19^. The dose-fractionated movies were aligned using the Patch Motion Correction, and the contrast transfer function (CTF) parameters were evaluated using the Patch-Based CTF estimation. Particles were automatically picked using Blob Picker and Template Picker, followed by reference-free 2D classification to curate particle sets. The particles were further curated by Heterogeneous Refinement (*N* = 5), using the map derived from the cryoSPARC Ab initio Reconstruction as a template. The best class containing 792,608 particles was refined using Homogeneous refinement followed by Non-uniform refinement^20^, yielding a map at 2.61 Å resolution. Local motion correction followed by Non-uniform refinement with optimization of the CTF value yielded a map at 2.55 Å resolution, according to the Fourier shell correlation (FSC) = 0.143 criterion^21^. The local resolution was estimated by BlocRes in cryoSPARC.

### Model building and validation

The initial model was built using Nautilus and Buccaneer^22^ in the CCP-EM package^23^ and manually built using COOT^24^ against the density map sharpened using DeepEMhancer^25^. The model was refined using Real-space refinement in PHENIX^26^ with the secondary structure, rotamer, and Ramachandran restraints. The structure was validated using MolProbity^27^ from the PHENIX package. The statistics of the 3D reconstruction and model refinement are summarized in Supplementary Table 1. The cryo-EM density maps were calculated with UCSF ChimeraX^28^, and molecular graphics figures were prepared with CueMol (http://www.cuemol.org).

### In vitro DNA cleavage assay

For in vitro DNA cleavage assays, the IscB–ωRNA complexes (WT or mutants) were expressed in *E. coli* and purified using Ni-NTA resin, in a similar manner to that for the complex prepared for the cryo-EM analysis (Supplementary Table 2). The IscB–ωRNA complex was applied to a RESOURCE Q column (GE Healthcare), equilibrated with buffer D (20 mM HEPES-NaOH, pH 7.5, 0.1 M NaCl, 10% glycerol, 5 mM MgCl_2_, and 2 mM DTT). The bound IscB–ωRNA complex was eluted with a linear gradient of 0.1–2 M NaCl. The peak fractions were collected, concentrated, and stored at −80 °C until use. Protein concentrations were determined using the Pierce 660 nm Protein Assay Reagent (Thermo Fisher Scientific).

To examine the structural integrity, the IscB–ωRNA complexes (WT or mutants), which were purified by chromatography on NiNTA and RESOURCE Q columns, were analyzed by size-exclusion chromatography. The IscB–ωRNA complex was diluted to 350 nM (IscB protein) with buffer E (20 mM Tris-HCl, pH 8.0, 0.5 M NaCl, 10% glycerol, 5 mM MgCl_2_, and 2 mM DTT), and then analyzed using a Superdex 200 10/300 Increase column, equilibrated with buffer E, in a fluorescence detection HPLC system (Shimadzu). The elution was monitored by the absorbances at 280 nm and 260 nm.

For in vitro DNA cleavage assays, a target double-stranded DNA (dsDNA), in which the TS and NTS were labeled with Cy5 and FAM, respectively, was prepared by PCR, using oligonucleotides listed in Supplementary Table 2. The 79-bp dsDNA template containing a 16-nt target sequence with a CTAG TAM was prepared by annealing the two oligonucleotides. The 150-bp target dsDNA was prepared by PCR, using the dsDNA template and the fluorescently labeled primers. The target DNA substrate (100 ng) was incubated with the purified IscB–ωRNA complex (250 nM protein) at 37 °C for 1 h, in 10 μL reaction buffer (20 mM HEPES-NaOH, pH 7.5, 60 mM NaCl, 5 mM MgCl_2_, 2% glycerol, and 0.4 mM DTT). The reaction solution was mixed with RNase A (NEB) and Proteinase K (Nacalai Tesque), boiled at 95 °C for 3 min with denaturing buffer (7 M urea), and then analyzed on a 10% Novex PAGE Tris–borate–EDTA (TBE)–urea gel (Invitrogen). The gels were imaged with a FUSION Solo S system, and the TS and NTS were visualized by the Cy5 and FAM fluorescence, respectively.

### Mammalian cell culture and transfection

Mammalian cell culture experiments were performed in the HEK293FT line (American Type Culture Collection (ATCC)) grown in Dulbecco’s Modified Eagle Medium with high glucose, sodium pyruvate, and GlutaMAX (Thermo Fisher Scientific), additionally supplemented with 1× penicillin–streptomycin (Thermo Fisher Scientific), 10 mM HEPES (Thermo Fisher Scientific), and 10% fetal bovine serum (VWR Seradigm). All cells were maintained at confluency below 80%. All transfections were performed with Lipofectamine 3000 (Thermo Fisher Scientific). Cells were plated 16–20 h prior to transfection, to ensure 90% confluency at the time of transfection. For 96-well plates, cells were plated at 2 × 10^4^ cells/well. For each condition, transfection plasmids were combined with OptiMEM I Reduced Serum Medium (Thermo Fisher Scientific) and 2 µL P3000 reagent per 1 µg of DNA, to a total volume of 25 µL. Separately, 23 µL of OptiMEM was combined with 2 µL of Lipofectamine 3000. The plasmid and Lipofectamine solutions were then combined, and 10 µL of the mixture was pipetted into each well.

### Mammalian genome editing assays

The IscB protein expression and ωRNA expression plasmids (200 ng each) were co-transfected into the wells of 96-well plates, as described^7^ (Supplementary Table 2). After 60–72 h, genomic DNA was harvested by washing the cells once with 1×DPBS (Sigma Aldrich) and adding 50 µL QuickExtract DNA Extraction Solution (Lucigen). Cells were scraped from the plates, suspended in QuickExtract, and cycled at 65 °C for 15 min, 68 °C for 15 min, and then 95 °C for 10 min for lysis. As input for each PCR reaction, 2.5 µL portions of cell lysates were used. For library amplification, target genomic regions were amplified by 12-cycles of PCR using NEBNext High Fidelity 2X PCR Master Mix (NEB), with an annealing temperature of 63 °C for 15 s, followed by a second 18-cycle round of PCR to add Illumina adapters and barcodes. The libraries were gel extracted and subjected to single-end sequencing on an Illumina MiSeq with Read 1300 cycles, Index 1 8 cycles, and Index 2 8 cycles. Insertion/deletion (indel) frequency was analyzed using CRISPResso2^29^. To eliminate noise from PCR and sequencing errors, only indels with at least 2 reads or >1 base inserted or 2 bases deleted were counted towards reported indel frequencies.

### Reporting summary

Further information on research design is available in the Nature Research Reporting Summary linked to this article.

## Supplementary information

Supplementary Information

Peer Review File

Description of Additional Supplementary Files

Supplementary Movie 1

Supplementary Movie 2

Supplementary Movie 3

Reporting Summary

## Data Availability

The structural model has been deposited in the Protein Data Bank under the accession code 7XHT. The EM density map has been deposited in the Electron Microscopy Data Bank under the accession code EMD-33586. Source data are provided with this paper.

## Source data

Source Data
