# Supplementary material for: Structure of the IscB–ωRNA ribonucleoprotein complex, the likely ancestor of CRISPR-Cas9
Source: Nat Commun. 2022 Nov 7;13:6719. doi: 10.1038/s41467-022-34378-3 (PMC9640706; doi:10.1038/s41467-022-34378-3)
Supplement: Supplementary file 1 — Supplementary Information [file 41467_2022_34378_MOESM1_ESM.pdf]

Structure of the IscB–ωRNA ribonucleoprotein complex, the likely ancestor of CRISPR-Cas9

Kato, Okazaki et al.

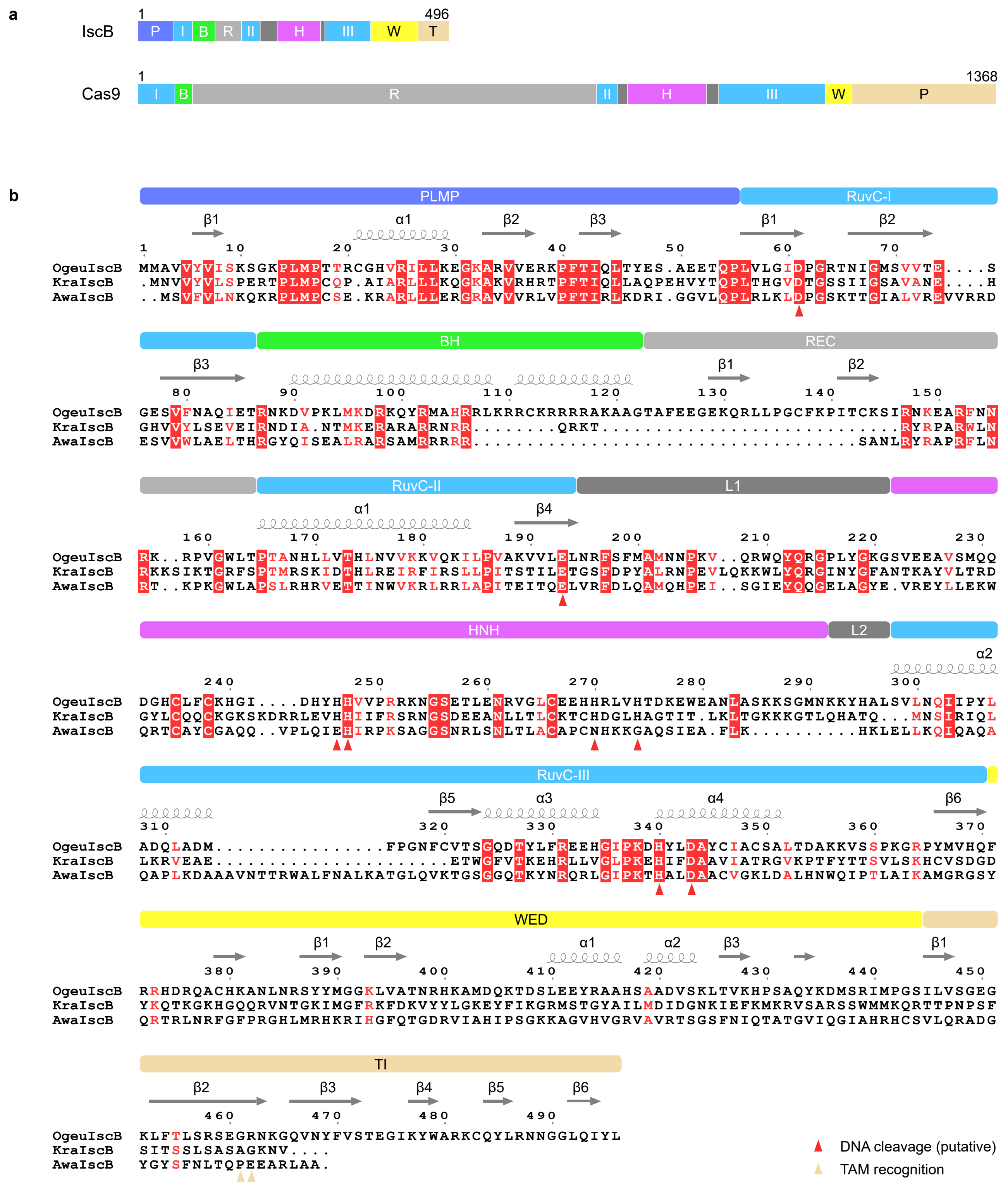

Supplementary Figure 1 | Domain organization.
a Domain structures of OgeuIscB and SpCas9. P, PLMP; I–III, RuvC-I–III; B, bridge helix; R, REC; H, HNH; W, WED; T, TI; P, PI.
b Multiple sequence alignment of the IscB proteins. OgeuIscB, IscB from the human gut metagenome (OGEU01000025.1); KraIscB, IscB from *Ktedonobacter racemifer* (ADVG01000004.1); AwaIscB, IscB from *Allochromatium warmingii* (FNOW01000019.1). The figure was prepared using Clustal Omega (http://www.ebi.ac.uk/Tools/msa/clustalo) and ESPript3 (http://espript.ibcp.fr/ESPript/ESPript).

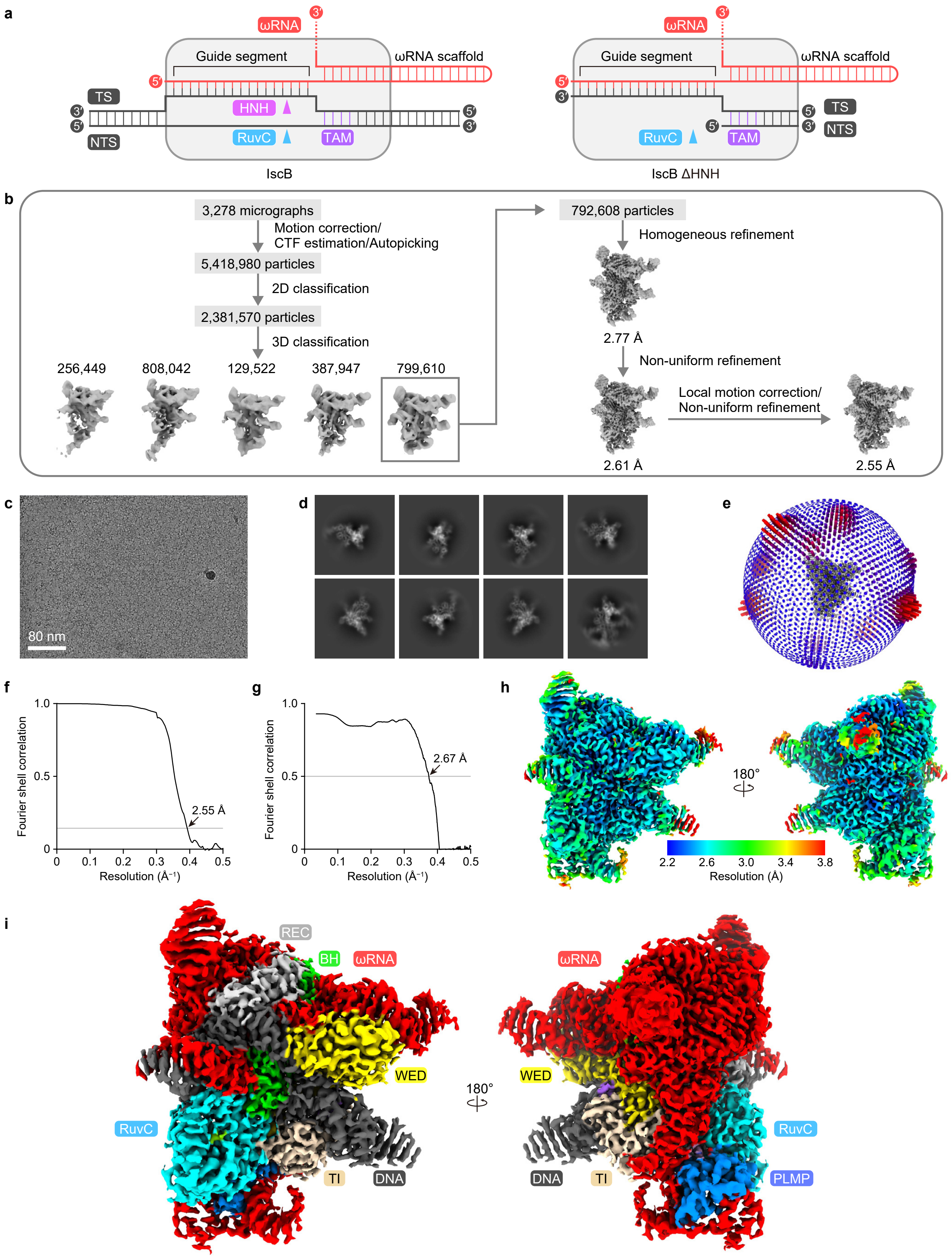

Supplementary Figure 2 | Cryo-EM analysis.

a Schematic of the ωRNA-guided DNA cleavage by IscB and the IscB–ωRNA–target DNA complex used for the cryo-EM analysis. The IscB ΔHNH mutant, in which residues 199–295 were substituted with GGSG, was used for the cryo-EM analysis. The IscB ΔHNH mutant contains the RuvC catalytic residues (D61, E193, and D343).

b Single-particle cryo-EM image processing workflow.

c Representative micrograph at a magnification of ×105,000.

d Representative 2D averaged class images from the particles used for final reconstruction.

e Euler angle distribution of particles in the final reconstruction.

f Fourier shell correlation curve calculated between the half-maps in the 3D reconstruction.

g Fourier shell correlation curve calculated between the refined model and the density map.

h, **i** Cryo-EM density maps according to the local resolution (**h**) and the structural domains (**i**).

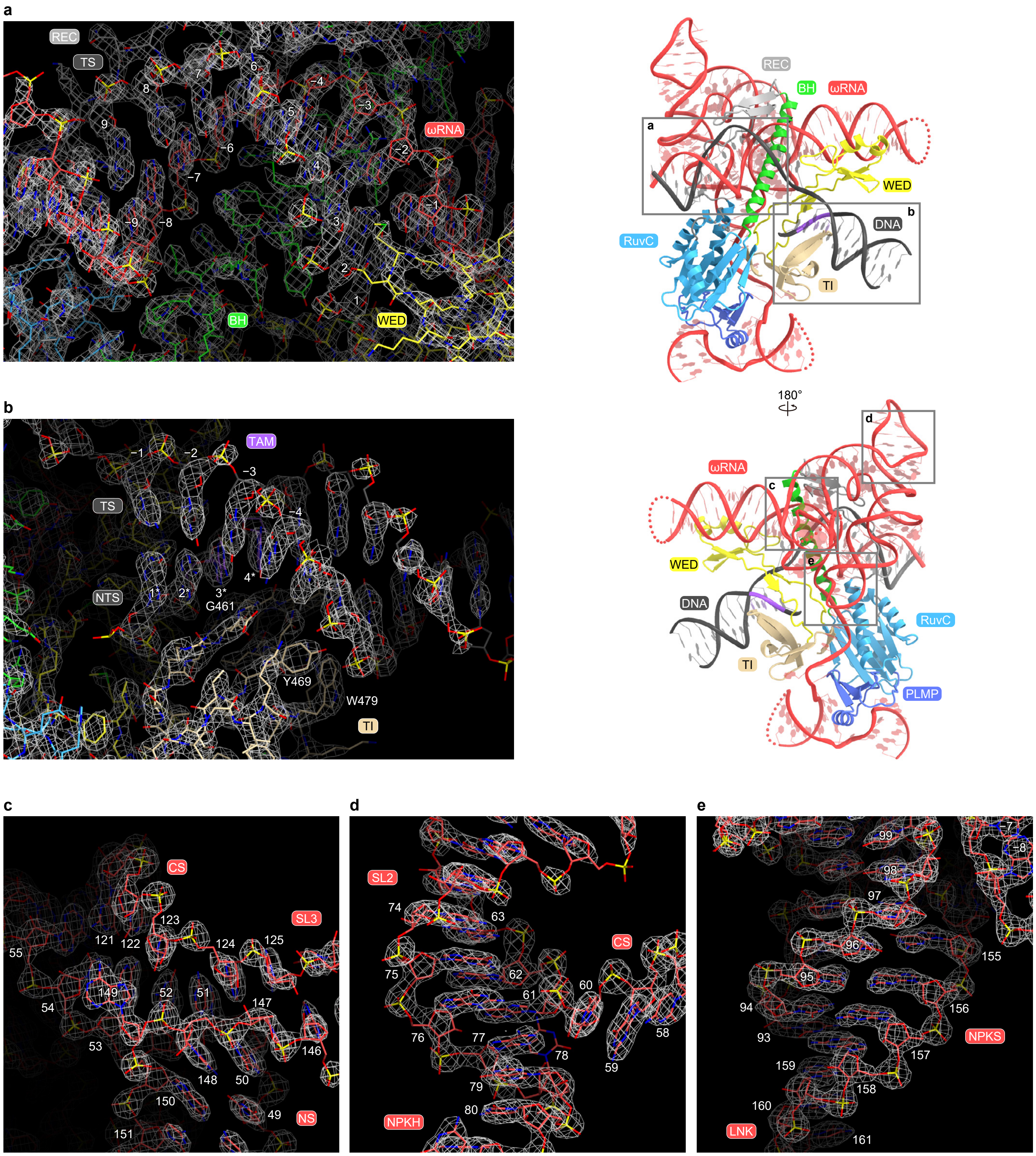

Supplementary Figure 3 | Cryo-EM density map.

Cryo-EM density maps for the guide RNA–target DNA heteroduplex (**a**), the TAM DNA duplex (**b**), and the ωRNA scaffold (**c**–**e**).

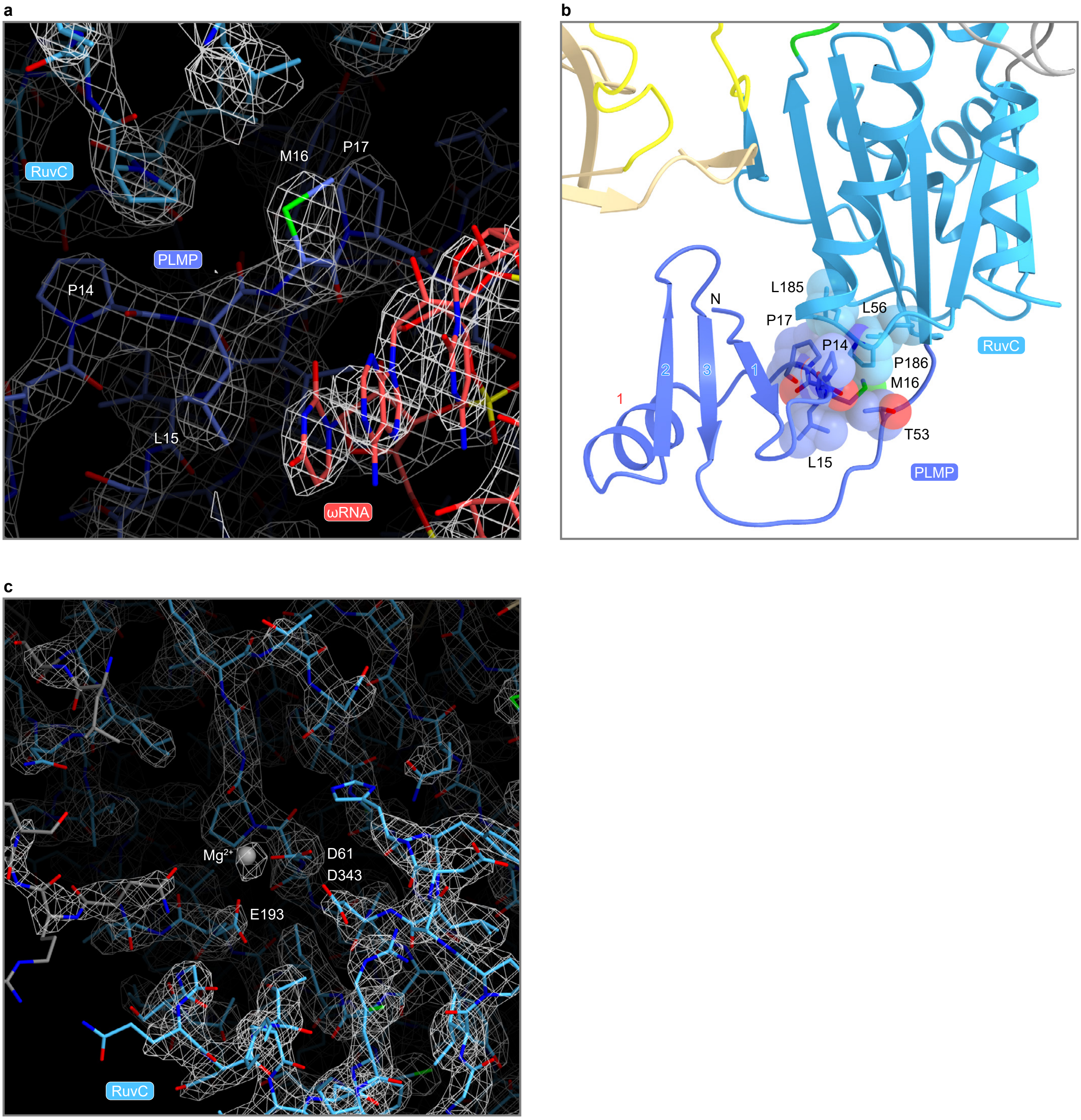

Supplementary Figure 4 | Structure of the PLMP and RuvC domains.

a Cryo-EM density map of the PLMP motif.

b Interactions between the PLMP and RuvC domains. Residues at the PLMP–RuvC interface are depicted as space-filling models.

c Cryo-EM density map of the RuvC active site. The bound Mg^{2+} ion is depicted as a gray sphere.

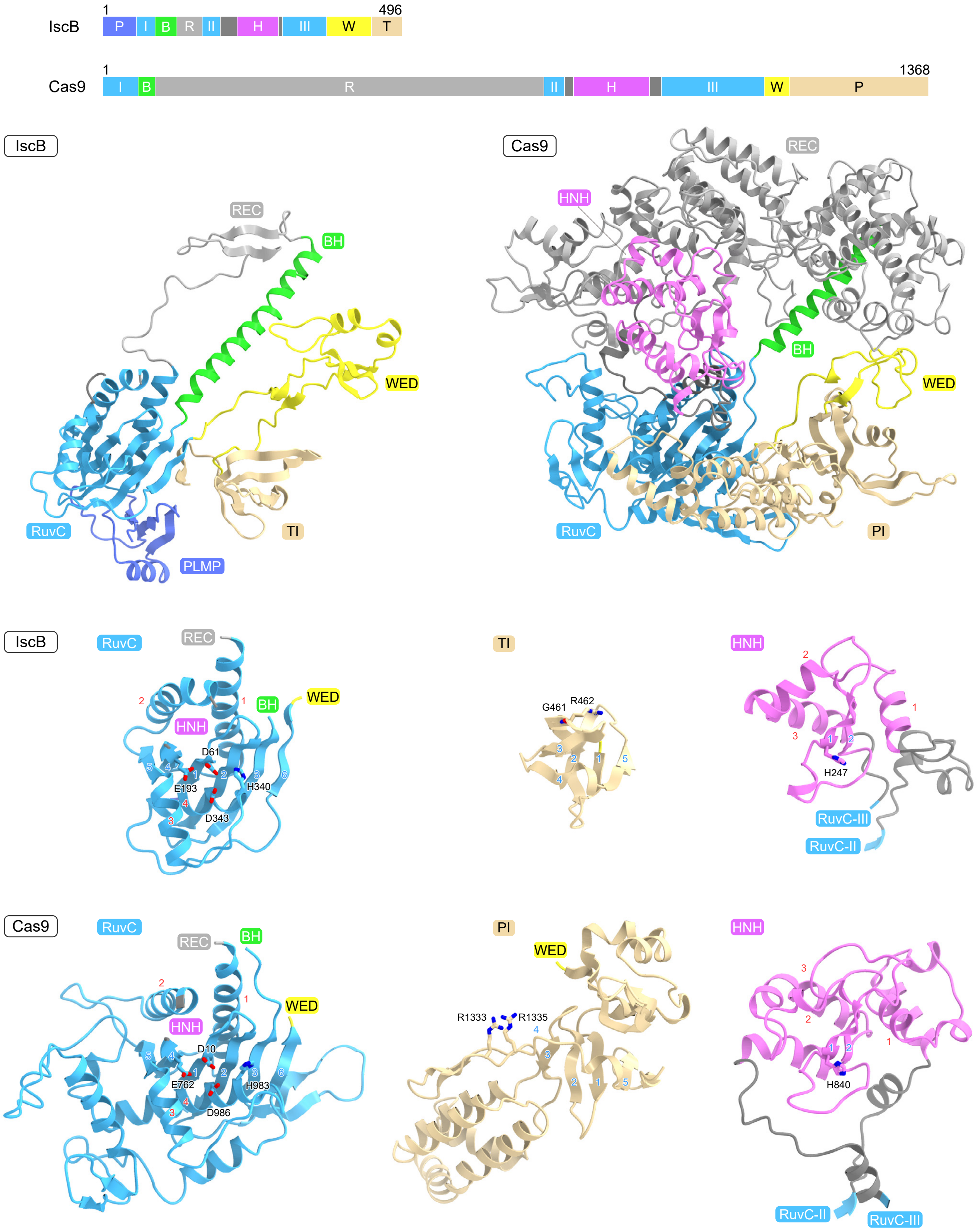

Supplementary Figure 5 | Structural comparison between IscB and Cas9.
The structures of IscB and SpCas9 (PDB: 5F9R) were aligned, based on their RuvC domains. The HNH domain of IscB was predicted by AlphaFold2. The catalytic residues in the RuvC and HNH domains and the TAM/PAM-interacting residues in the TI/PI domains are shown as stick models. The core α-helices and β-strands in each domain are numbered in red and blue, respectively.

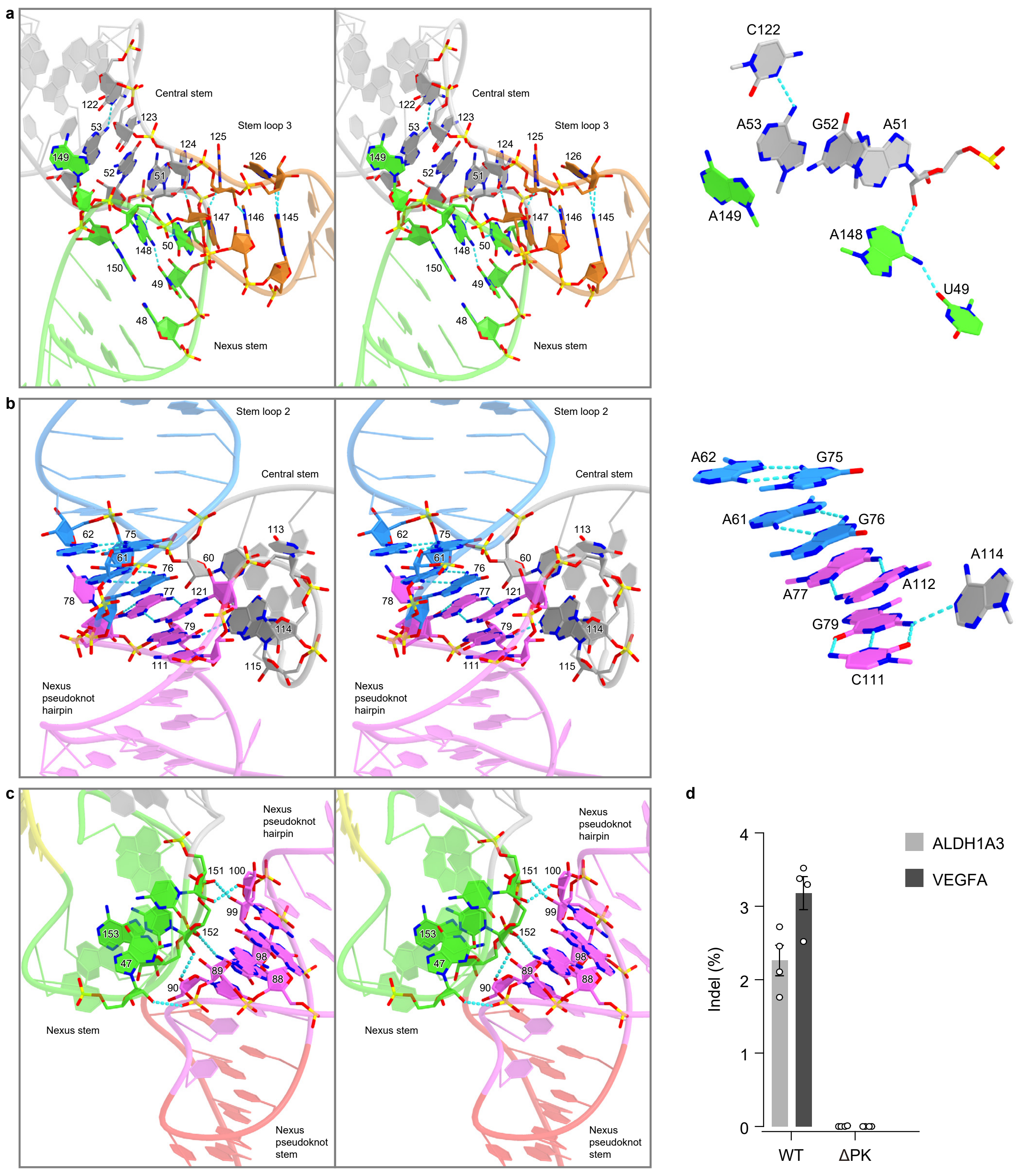

Supplementary Figure 6 | ωRNA architecture.

a–**c** Stereo views of the three-way junction (nexus stem, central stem, and stem loop 3) (**a**), the three-way junction (central stem, stem loop 2, and nexus pseudoknot hairpin) (**b**), and the interface between the nexus stem and the nexus pseudoknot hairpin (**c**). In (**a**), N6 of A148 hydrogen bonds with O2 of U49, forming a non-canonical A-U base pair. In (**a**) and (**b**), close-up views of the key interactions are shown on the right of the stereo views.

d Indel activities of the wild-type (WT) IscB in complex with the WT ωRNA or the ΔPK ωRNA mutant, in which nucleotides C93–C96 were replaced with GGGG. Data are mean ± s.e.m. (n = 4, biologically independent samples). The experiments were repeated four times with similar results. Source data are provided at the end of the Supplementary Information.

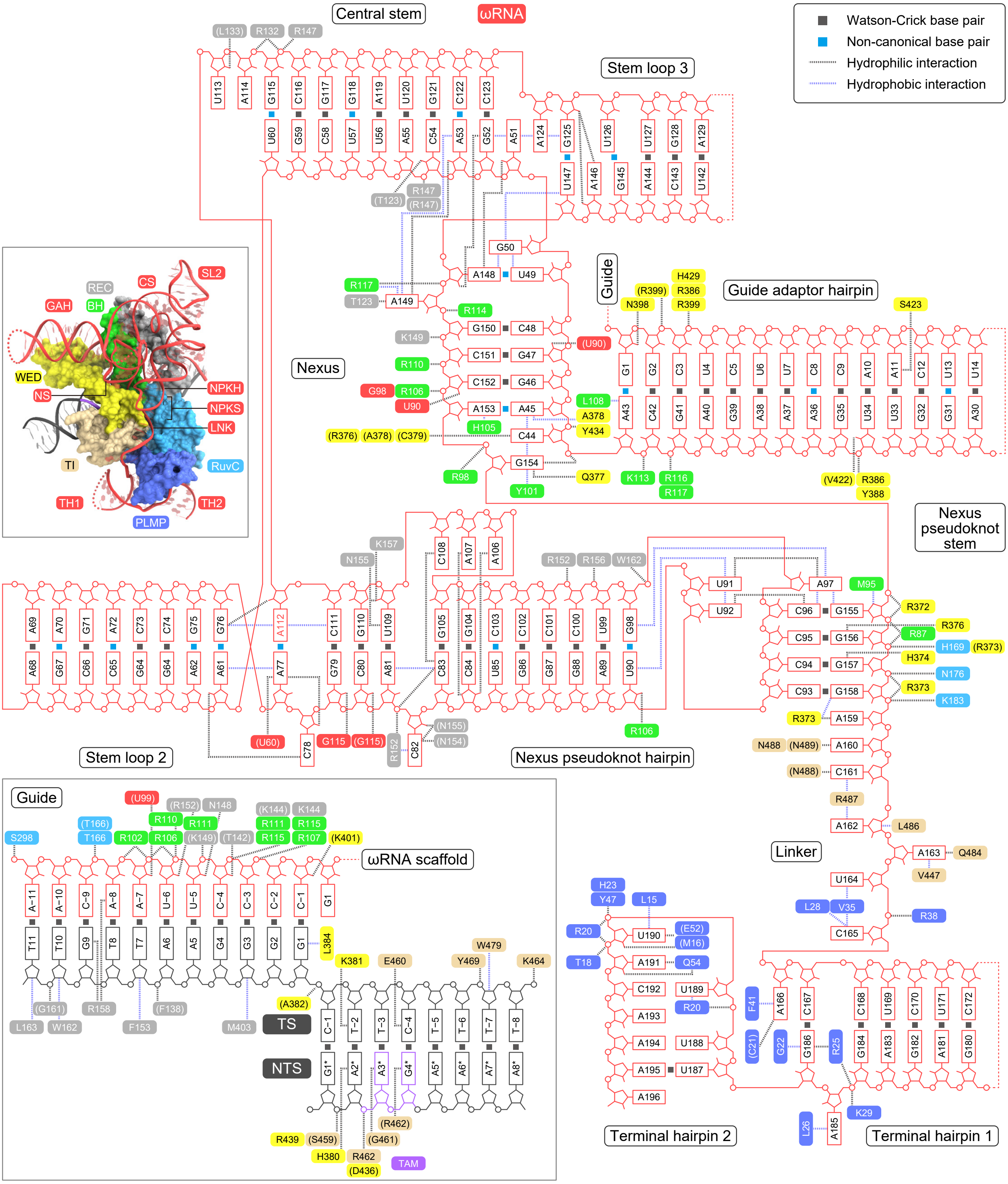

Supplementary Figure 7 | Schematic of the IscB–ωRNA–DNA interactions.
A112 (labeled in red) adopts the *syn* conformation.

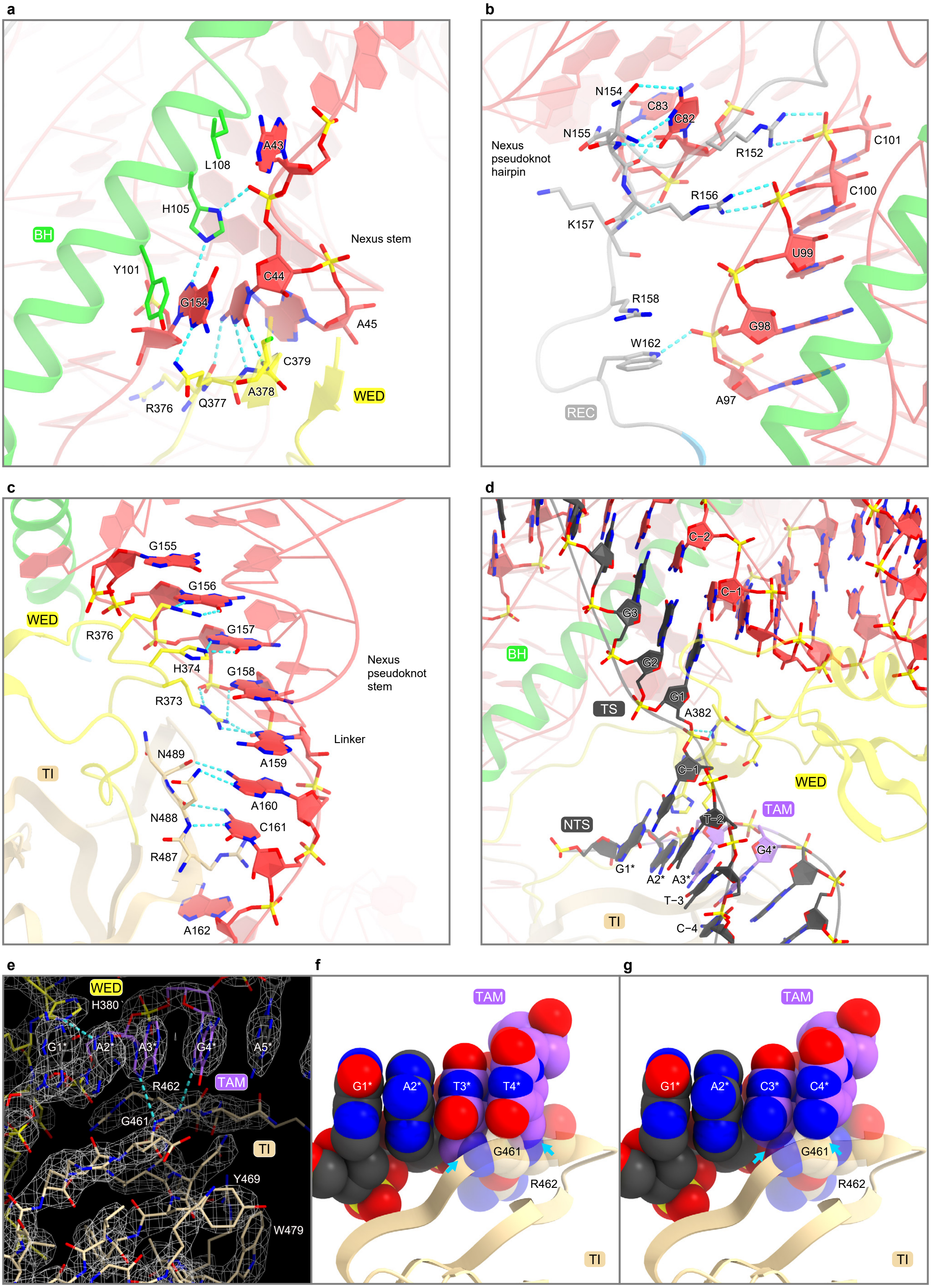

Supplementary Figure 8 | IscB–ωRNA–DNA interactions.

a–**d** Recognition of the nexus stem (**a**), the nexus pseudoknot hairpin (**b**), the nexus pseudoknot stem and linker (**c**), and the TAM duplex (**d**).

e Cryo-EM density map of the TI domain and the TAM. Hydrogen bonds are indicated by dashed lines.

f, **g** Manual modeling of T (**f**) and C (**g**) nucleotides at positions 3 and 4 in the NNRR TAM. The TAM nucleotides and G461/R462 are depicted by space-filling models. Possible steric clashes between the modeled T/C bases and G461/R462 are indicated by cyan arrows.

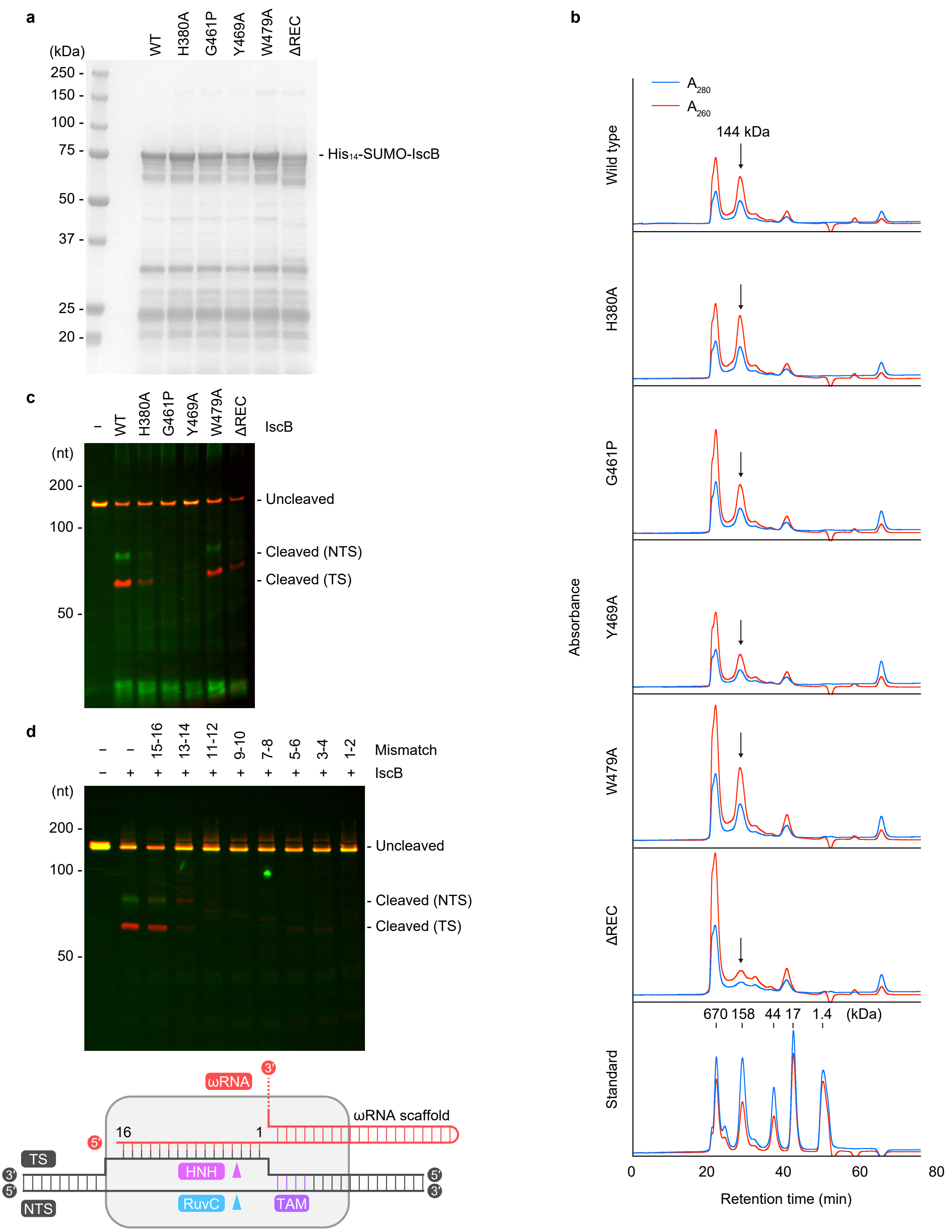

Supplementary Figure 9 | *In vitro* DNA cleavage experiments.

a SDS-PAGE analysis of the IscB–ωRNA complexes (WT and mutants) used for *in vitro* DNA cleavage experiments. The IscB–ωRNA complexes were purified on NiNTA and RESOURCE Q columns.

b Size-exclusion chromatography analysis of the purified IscB–ωRNA complexes (WT and mutants). The IscB–ωRNA complexes were purified by chromatography on NiNTA and RESOURCE Q columns, and then aliquots of the peak fractions from the RESOURCE Q step were analyzed on a Superdex 200 10/300 Increase column. The active fractions are indicated by arrows. Like the WT, the H380A, G461P, Y469A, and W479A mutants eluted from the gel-filtration column as symmetrical peaks, indicating that these mutations do not substantially affect the overall structures. In contrast, the peak fraction of the ΔREC mutant was relatively smaller, suggesting the importance of the REC linker for maintaining the structural integrity of the IscB–ωRNA complex.

c *In vitro* DNA cleavage activities of WT and mutant IscBs. The 150-bp double-stranded target DNA was incubated with the IscB–ωRNA complex (WT or mutants) at 37°C for 1 h, and then the reaction was analyzed using a 10% TBE–urea gel.

d Effects of mismatches between the ωRNA guide and the target DNA on IscB-mediated DNA cleavage. The 150-bp double-stranded target DNA (containing no mismatch or 2-nt mismatches at positions 1–16) was incubated with the WT IscB–ωRNA complex at 37°C for 1 h, and then the reaction was analyzed by 10% denaturing urea-PAGE. In (**c**) and (**d**), the target DNA strand (TS) and non-target DNA strand (NTS) were visualized, using Cy5 and FAM fluorescence, respectively.

The experiments were repeated at least three times with similar results. Source data are provided at the end of the Supplementary Information.

Supplementary Table 1 | Cryo-EM data collection, refinement, and validation statistics.

	OgeuIscB (EMDB-33198) (PDB 7XHT)
Data collection and processing	
Magnification	105,000
Voltage (kV)	300
Electron exposure (e⁻/Å²)	47.9
Defocus range (μm)	−0.8 to −2.0
Pixel size (Å)	0.83
Symmetry imposed	*C*1
Initial particle images (no.)	5,418,980
Final particle images (no.)	792,405
Map resolution (Å)	2.55
FSC threshold	0.143
Refinement	
Model resolution (Å)	2.67
FSC threshold	0.5
Map sharpening *B* factor (Å²)	92.6
Model composition	
Non-hydrogen atoms	7839
Protein residues	395
Nucleotide residues	220
Ligands	3
B factors (Å²)	
Protein	27.37
Nucleotide	24.88
Ligand	35.73
R.m.s. deviations	
Bond lengths (Å)	0.003
Bond angles (°)	0.539
Validation	
MolProbity score	1.34
Clashscore	6.14
Poor rotamers (%)	0.00
Ramachandran plot	
Favored (%)	98.21
Allowed (%)	1.79
Disallowed (%)	0.00

Supplementary Table 2 | Nucleotide sequences used in this study.

Cryo-EM analysis

Name	Sequence
ωRNA	GGAAUUGUGAGCGGAUAACAAUUCCCCGGCUCUUCCAACUUUAUGGUUGCGACCGUAGGUUGAAAGAGCACAGGCUGAG ACAUUCGUAAGGCCGAAAGACCGGACGCACCCUGGGAUUUCCCCAGUCCCCGGAACUGCAUAGCGGAUGCCAGUUGAUG GAGCAAUCUAUCAGAUAAGCCAGGGGGAACAAUCACCUCUCUGUAUCAGAGAGAGUUUUACAAAAGGAGGAACGG
TS	GAATGGTTTTCTTCGGGGAATTGTTATCCGCTCACAATTCCTTAGAAAA
NTS	GAAGAAAACCATTC

The guide sequence in the ωRNA and its complementary sequence in the TS are underlined. The TAM sequence (GAAG) in the NTS is colored purple.

pETDuet-OgeuIscB_1	https://benchling.com/s/seq-fj7MExHIBYNTgn7gRgQV?m=slm-VJtf6i7HfXSztOdwlYih

***In vitro* DNA cleavage assays**

Name	Sequence
On-target_F	CCCACGAAGGGTTACGGCACCTTAACCCCTTATTAGGTGCGCTTGGCCTAGAAGTCTGAAAAGGTCATTTTTTAAAGCC
MM12_F	CCCACGAAGGGTTACGGCACCTTAACCCCTTATTAGGTGCGCTTGcgCTAGAAGTCTGAAAAGGTCATTTTTTAAAGCC
MM34_F	CCCACGAAGGGTTACGGCACCTTAACCCCTTATTAGGTGCGCTacGCCTAGAAGTCTGAAAAGGTCATTTTTTAAAGCC
MM56_F	CCCACGAAGGGTTACGGCACCTTAACCCCTTATTAGGTGCGgaTGGCCTAGAAGTCTGAAAAGGTCATTTTTTAAAGCC
MM78_F	CCCACGAAGGGTTACGGCACCTTAACCCCTTATTAGGTGgcCTTGGCCTAGAAGTCTGAAAAGGTCATTTTTTAAAGCC
MM910_F	CCCACGAAGGGTTACGGCACCTTAACCCCTTATTAGGacCGCTTGGCCTAGAAGTCTGAAAAGGTCATTTTTTAAAGCC
MM1112_F	CCCACGAAGGGTTACGGCACCTTAACCCCTTATTAccTGCGCTTGGCCTAGAAGTCTGAAAAGGTCATTTTTTAAAGCC
MM1314_F	CCCACGAAGGGTTACGGCACCTTAACCCCTTATatGGTGCGCTTGGCCTAGAAGTCTGAAAAGGTCATTTTTTAAAGCC
MM1516_F	CCCACGAAGGGTTACGGCACCTTAACCCCTTtaTAGGTGCGCTTGGCCTAGAAGTCTGAAAAGGTCATTTTTTAAAGCC
On-target_R	GGCTTTAAAAAATGACCTTTTCAGACTTCTAGGCCAAGCGCACCTAATAAGGGGTTAAGGTGCCGTAACCCTTCGTGGG
MM12_R	GGCTTTAAAAAATGACCTTTTCAGACTTCTAGcgCAAGCGCACCTAATAAGGGGTTAAGGTGCCGTAACCCTTCGTGGG
MM34_R	GGCTTTAAAAAATGACCTTTTCAGACTTCTAGGCgtAGCGCACCTAATAAGGGGTTAAGGTGCCGTAACCCTTCGTGGG
MM56_R	GGCTTTAAAAAATGACCTTTTCAGACTTCTAGGCCAtcCGCACCTAATAAGGGGTTAAGGTGCCGTAACCCTTCGTGGG
MM78_R	GGCTTTAAAAAATGACCTTTTCAGACTTCTAGGCCAAGgcCACCTAATAAGGGGTTAAGGTGCCGTAACCCTTCGTGGG
MM910_R	GGCTTTAAAAAATGACCTTTTCAGACTTCTAGGCCAAGCGgtCCTAATAAGGGGTTAAGGTGCCGTAACCCTTCGTGGG
MM1112_R	GGCTTTAAAAAATGACCTTTTCAGACTTCTAGGCCAAGCGCAggTAATAAGGGGTTAAGGTGCCGTAACCCTTCGTGGG
MM1314_R	GGCTTTAAAAAATGACCTTTTCAGACTTCTAGGCCAAGCGCACCatATAAGGGGTTAAGGTGCCGTAACCCTTCGTGGG
MM1516_R	GGCTTTAAAAAATGACCTTTTCAGACTTCTAGGCCAAGCGCACCTAtaAAGGGGTTAAGGTGCCGTAACCCTTCGTGGG
FAM_F	FAM-CCGCAAGAGGATGATTCGGGTGCGGCAACGGAAGGGGAGGGCCCCACGAAGGGTTACGG
Cy5_R	Cy5-GCTGATCTGATGCAGTTAAGTGCCTGCTGGGCTTTAAAAAATGACCTTTTCAGAC

The target sequences are underlined. Mismatches are shown in lowercase. The TAM sequences (CTAG) are colored purple.

pETDuet-OgeuIscB_2	https://benchling.com/s/seq-1R6enujAzTL44kexhfiM?m=slm-XsK2idX0THkGW0HttP4R

Genome editing assays

Guides

Target	Guide sequence
ALDH1A3	AGTGGAAGAAGGAGAT
VEGFA	AAAAGAGTGAACGAGA
Non-targeting	GTCGACGCATAGTCTG

NGS round 1 PCR primers

Target	Primer sequence
ALDH1A3_F	CTTTCCCTACACGACGCTCTTCCGATCTCGGCACGAATCCAAGAGTGGGAAAAAG
ALDH1A3_R	GACTGGAGTTCAGACGTGTGCTCTTCCGATCGCCATATGATGAGGATAGCTGAGGTCATC
VEGFA_F	CTTTCCCTACACGACGCTCTTCCGATCTCGGCCCTCCTCTGTCCCAATTGT
VEGFA_R	GACTGGAGTTCAGACGTGTGCTCTTCCGATCCCCTCCAAGGTCTGCTTTCCAGA

Primer-binding sequences are underlined.

Source data: Raw data for Supplementary Figure 6d: Indel activity

Table format: Grouped		Group A ALDH1A3				Group B VEGFA			
		A:Y1	A:Y2	A:Y3	A:Y4	B:Y1	B:Y2	B:Y3	B:Y4
1	WT	2.72	2.11	1.76	2.46	3.30	3.38	3.52	2.52
2	E129-K144 deletion	0.01	0.00	0.01	0.00	0.00	0.02	0.02	0.00
3	H380A	0.84	0.74	1.08	0.93	0.43	0.28	0.17	0.31
4	G461P	0.00	0.00	0.00	0.00	0.00	0.00	0.00	0.02
5	Y469A	0.15	0.16	0.15	0.25	0.00	0.07	0.02	0.00
6	W479A	2.83	1.01	2.23	2.68	0.84	1.04	1.26	0.84
7	93-CCCC-96 -> GGGG	0.00	0.00	0.00	0.01	0.00	0.00	0.00	0.00

Supplementary Figure 6d

Source data: Uncropped gels for Supplementary Figure 9

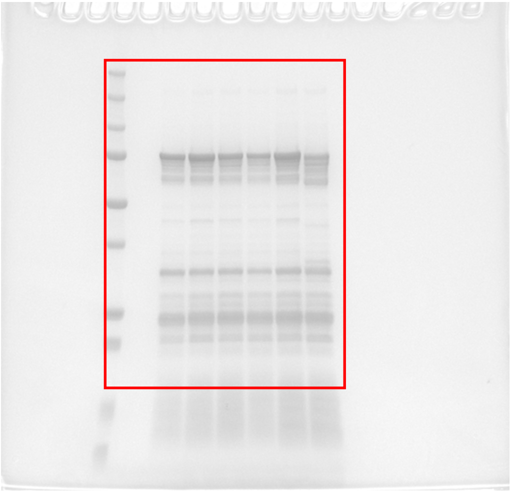

Supplementary Figure 9a

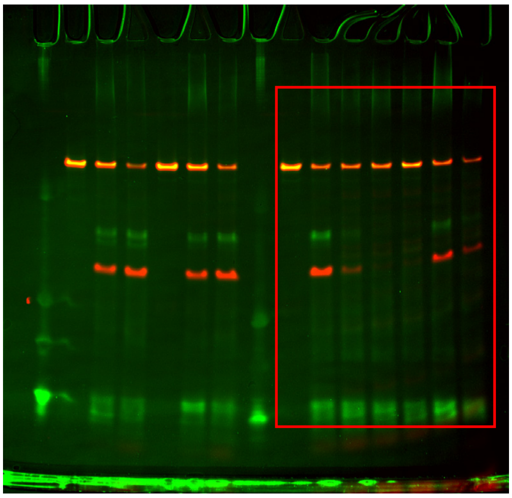

Supplementary Figure 9c

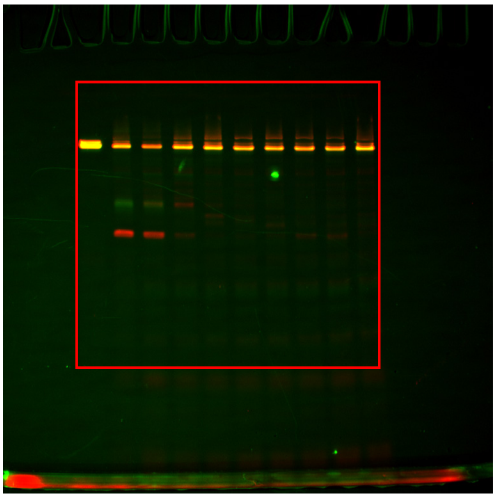

Supplementary Figure 9d
