# Supplementary material for: Structure of the IscB–ωRNA ribonucleoprotein complex, the likely ancestor of CRISPR-Cas9
Source: Nat Commun. 2022 Nov 7;13:6719. doi: 10.1038/s41467-022-34378-3 (PMC9640706; doi:10.1038/s41467-022-34378-3)
Supplement: Supplementary file 3 — Description of Additional Supplementary Files [file 41467_2022_34378_MOESM3_ESM.pdf]

Description of Additional Supplementary Files

File Name: Supplementary Movie 1
Description: Cryo-EM density of the IscB–ωRNA–DNA complex.

File Name: Supplementary Movie 2
Description: Structure of the IscB–ωRNA–DNA complex.

File Name: Supplementary Movie 3
Description: Structure of the ωRNA.
